# DNA methylation-based forensic age prediction using artificial neural networks and next generation sequencing

**DOI:** 10.1016/j.fsigen.2017.02.009

**Published:** 2017-05

**Authors:** Athina Vidaki, David Ballard, Anastasia Aliferi, Thomas H. Miller, Leon P. Barron, Denise Syndercombe Court

**Affiliations:** Department of Pharmacy and Forensic Science, King’s College London, Franklin-Wilkins Building, 150 Stamford Street, London, UK

**Keywords:** Forensic epigenetics, Chronological age prediction, DNA methylation, Next generation sequencing, Artificial neural networks

## Abstract

The ability to estimate the age of the donor from recovered biological material at a crime scene can be of substantial value in forensic investigations. Aging can be complex and is associated with various molecular modifications in cells that accumulate over a person’s lifetime including epigenetic patterns. The aim of this study was to use age-specific DNA methylation patterns to generate an accurate model for the prediction of chronological age using data from whole blood. In total, 45 age-associated CpG sites were selected based on their reported age coefficients in a previous extensive study and investigated using publicly available methylation data obtained from 1156 whole blood samples (aged 2–90 years) analysed with Illumina’s genome-wide methylation platforms (27 K/450 K). Applying stepwise regression for variable selection, 23 of these CpG sites were identified that could significantly contribute to age prediction modelling and multiple regression analysis carried out with these markers provided an accurate prediction of age (R^2^ = 0.92, mean absolute error (MAE) = 4.6 years). However, applying machine learning, and more specifically a generalised regression neural network model, the age prediction significantly improved (R^2^ = 0.96) with a MAE = 3.3 years for the training set and 4.4 years for a blind test set of 231 cases. The machine learning approach used 16 CpG sites, located in 16 different genomic regions, with the top 3 predictors of age belonged to the genes NHLRC1, SCGN and CSNK1D. The proposed model was further tested using independent cohorts of 53 monozygotic twins (MAE = 7.1 years) and a cohort of 1011 disease state individuals (MAE = 7.2 years). Furthermore, we highlighted the age markers’ potential applicability in samples other than blood by predicting age with similar accuracy in 265 saliva samples (R^2^ = 0.96) with a MAE = 3.2 years (training set) and 4.0 years (blind test). In an attempt to create a sensitive and accurate age prediction test, a next generation sequencing (NGS)-based method able to quantify the methylation status of the selected 16 CpG sites was developed using the Illumina MiSeq^®^ platform. The method was validated using DNA standards of known methylation levels and the age prediction accuracy has been initially assessed in a set of 46 whole blood samples. Although the resulted prediction accuracy using the NGS data was lower compared to the original model (MAE = 7.5 years), it is expected that future optimization of our strategy to account for technical variation as well as increasing the sample size will improve both the prediction accuracy and reproducibility.

## Highlights

• Blood DNA methylation profiles of 1156 individuals were assessed for age correlation.
• Stepwise regression identified 23 age-associated CpG sites in DNA from blood.
• A machine learning model based on 16 markers predicted age with a mean error of 3.8 years.
• The model predicted age successfully for twins and ‘diseased’ individuals.
• A new NGS-based method was combined with machine learning for age prediction.

## 1 Introduction

Body fluids such as blood are amongst the most important biological evidence recovered from crime scenes. Identification of the donor can be achieved through short tandem repeat (STR) profiling; nevertheless, extracting additional information regarding the donor, such as chronological age, could provide significant investigative leads and prove very useful in police investigations. For intelligence purposes, estimating the age of a recovered stain’s donor could potentially narrow down the number of suspects, especially in cases where an eyewitness is not available.

Over the last decades, research has shown that aging is a very complex process influenced by various genetic, lifestyle and environmental factors. It causes a variety of molecular modifications and adjustments in tissues or organs that accumulate over an individual’s lifetime, including chemical modifications [1], gene expression alterations [2] and variations at the DNA level [3], [4]. Although there have been various approaches to estimate age at death in human remains or chronological age in living individuals [5], [6], most of these attempts show limitations including low sensitivity and prediction accuracy as well as lack of standardisation, restraining their applicability in crime scene samples. Undoubtedly, developing an age prediction test is a major challenge for forensic scientists since they would need to be able to apply and validate it using minute or degraded samples consisting of a range of tissues and body fluids. As a first step, the generation of reliable age prediction models is a necessity.

It is believed that epigenetic analysis could serve as an alternative or supplementary method to existing approaches since particularly DNA methylation is well-known to be one of the mechanisms responsible for cell differentiation and the cellular response to aging [7], [8]. It is generally suggested that there is an increase in global epigenetic drift with age [9] and various genome-wide methylation analyses have revealed a substantial decrease in global DNA methylation levels with advancing age [10]. Changes in DNA methylation patterns due to aging are quickly observed during the first months of an individual’s life and throughout childhood [11], [12]. Cumulative evidence points towards the distinct contributions of genetic [13], environmental [14], [15] and stochastic factors to DNA methylation levels at single genomic areas. In order to identify specific age-associated differentially methylated CpG sites for a particular body fluid, scientists have chosen to perform genome-wide studies [7], [16], [17], [18], [19], [20]. Interestingly, >95% of the associated sites were located within 500 bp of the transcriptional start site of the associated gene, implying a connection with regulation of gene expression [16].

From an intelligence perspective, it would be very advantageous to translate observed biological age-associated DNA methylation differences in a way that the chronological age of an individual is revealed through an age prediction model. Overall, current methodologies for methylation analysis can be divided into genome-wide or gene-specific depending on the number of CpG sites being investigated. As an example, following analysis of >650 whole blood samples from individuals aged 19–101 years using a genome-wide approach, Hannum et al. built a quantitative model using 71 highly age-predictive markers with a correlation between true and predicted age of 0.96 and an average error of 3.9 years [21]. However, it should be emphasised that each tissue or body fluid could show a different age-associated DNA methylation pattern; therefore, predicting age across a broad spectrum of human tissues and cell types could be a very challenging task. Testing 13 different cell types, Kock and Wagner [22] proposed a set of 5 CpG sites, however the precision of their model was slightly lower (mean error of 9.3 years). While the genome-wide DNA methylation arrays are considered as the best tool during the discovery phase of potential age-associated CpG sites, targeted sequencing is also required to validate any association. Replicating the detected methylation levels is necessary to confirm the utility of the selected CpG sites and assess their performance in a different dataset. From a forensic perspective, the main challenge to be faced is the low quality and quantity of forensic specimens, making it impossible to implement such age prediction models (based on hundreds of markers) in forensic casework in their current form. Therefore, developing an accurate, robust and sensitive method that can analyse the proposed CpG sites in forensic-type samples is essential.

In an attempt to narrow down the number of markers needed for accurate prediction, Weidner et al. [23] performed a comprehensive analysis of blood methylation profiles and found that the methylation levels of only 3 CpGs – located in the integrin, alpha 2b (*ITGA2B*), aspartoacylase (*ASPA*) and phosphodiesterase 4C, cAMP specific (*PDE4C*) genes – were substantial to create an epigenetic-aging-signature with a mean absolute deviation (MAD) from chronological age of 5.4 years (RMSE = 7.2 years). Within the forensic field, recent age prediction models based on a small number of CpG sites have also been studied, mainly in blood [24], [25], [26], [27], [28], [29], [30], but also in other tissues such as saliva [31], semen [32] and teeth [33]. However, most of these models are based on a limited number of individuals and some still lack validation in an independent cohort of samples. The reported mean prediction errors range between 4–8 years (especially in validation sets where available), suggesting that current tools allow for the prediction of an individual’s decade (for example, the blood belongs to someone in their 30s) rather than an accurate prediction outcome. In this study, in an attempt to minimise the prediction error and increase model accuracy, the potential of artificial neural networks (ANN) was explored together with regression analysis. ANNs are a group of machine learning algorithms inspired by biological systems and have previously been used successfully to find underlying trends in complex datasets. There are various types of ANNs and the best type to be used depends on the application. Normally, ANNs consist of discrete layers; the first is the input layer, which contains the dependent variables (i.e. methylation data from age-dependent CpG sites). Each of these variables are connected to a middle layer via an optimised number of ‘nodes’, which, in turn, interconnect all inputs to each other and eventually to the third layer containing the designated output variable (i.e. age in years). During the learning process, ANNs generally aim to minimise the error in output estimations by systematically optimising the connective weights between the nodes within the network. Given their ability to learn holistically and often in a non-linear fashion, ANNs have been extensively studied and applied in a range of other applications [34], [35].

Also, while most of these studies are based on targeted methylation detection via pyrosequencing, qPCR [36], melting curve analysis [37] and the EpiTYPER system [38], [39] have also been used. Although pyrosequencing has been the gold standard for such analysis since its introduction [40], [41] and shows various advantages over other methylation techniques [42], it is mainly performed as single reactions because multiplex pyrosequencing can be complex [43]. In this study we also address the question of whether a methylation assay based on benchtop next-generation sequencing (NGS) of a small number of CpG sites could not only provide focused 5-methylcytosine quantification with base resolution, but also allow for a sensitive and less costly age prediction approach with similar accuracy to genome-wide DNA methylation profiling approaches that could be applied in a forensic setting.

Towards achieving this aim, we pooled publicly available DNA methylation profiles derived from whole blood samples to investigate a subset of 45 previously reported age-associated CpGs, which belong to 45 different genomic locations/genes, in an attempt to identify those displaying the highest correlation with age. Following multivariate, linear regression and ANN analysis, we identified an epigenetic aging signature based on the methylation status of a total of 16 CpG sites. To allow for reliable age predictions, a next-generation sequencing protocol based on Illumina’s MiSeq^®^ platform was developed and optimised using commercially available DNA methylation standards. To the best of our knowledge, this is the first study that uses machine learning, via ANNs, together with an NGS-based DNA methylation detection method for forensic age prediction.

## 2 Materials and methods

### 2.1 Description of genome-wide DNA methylation data sets

Genome-wide profiling has led to a more comprehensive understanding of gene regulation epigenetic mechanisms. Illumina’s Human Methylation BeadChip technology is one of the most commonly used genome-wide methylation platforms that allows for simultaneous measurement of the methylation status of 27,578 (27 K chip) or 482,421 (450 K chip) CpG sites in the genome at single nucleotide resolution. Thousands of samples have been assayed using this platform in the literature and researchers have made some of these genome-wide methylation data available in online databases such as the National Center for Biotechnology Information Gene Expression Omnibus (GEO).

In order to build the age prediction model, data from a total of 1156 whole blood samples were collected from individuals aged between 2 and 90 years old and from various ethnic backgrounds (mean age = 44) from seven genome-wide DNA methylation studies summarised in Table S1 [12], [16], [17], [19], [21], [44]. Methylation data gathered from individual blood cell types such as peripheral blood mononuclear cells (PBMCs) or CD4+ cells were avoided since the ultimate aim of this research was to predict age from whole blood stains. Samples were carefully collected so that there was an equal representation of samples for all age groups, aiming for ∼100–150 samples per decade (see Fig. S1). The gathered samples were either healthy control volunteers in studies investigating DNA methylation changes of various diseases (usually above 40 years old) or were part of studies investigating epigenetic effects of aging (usually either very young or very old), hence collecting sufficient samples of ‘middle’ age (particularly 30–40 years old) was quite challenging. Additionally, even though the dataset included roughly equal numbers of both females and males (597 and 559 respectively), there was an uneven gender distribution within specific age groups due to the selected studies’ design (Fig. S1). However, it was concluded that this should not affect age prediction since none of the sex-specific differentially methylated CpG sites previously reported in the literature, following analysis with Illumina’s 27 K platform, were included in the group of selected markers in this study [44].

Following the development of age prediction models, environmental influences on age prediction were further investigated using an independent cohort of healthy blood samples comprising of 53 female monozygotic twin pairs collected from two genome-wide studies [7], [45] (Table S1). Secondly, to test the robustness of the selected age-associated CpG sites when applied to body fluids other than blood, methylation data from 265 saliva samples was collected from two different studies [46], [47] (Table S1). One key limitation when building a model for body fluids other than blood is the scarcity of non-blood based genome-wide studies that are both run on one of the Illumina platforms and include information regarding the volunteers’ age. Finally, according to Horvath [48] the correlation between the observed and expected age in cancer/diseased tissues was generally weak as there was evidence of significant biological age acceleration in most patients included in his study (n = 5826). However, since there is usually no information regarding possible disease status in a forensic blood sample of unknown origin, it is important that the proposed age prediction model can be universally applied. To assess potential variability in age prediction, a data set including blood samples from a total of 1011 (577 females and 434 males) individuals aged 17–91 years suffering from various diseases and cancers analysed on Illumina’s 27 K or 450 K platforms was analysed [8], [17], [19], [49], [50], [51], [52] (Table S2).

In each dataset, the DNA methylation value of each CpG site is calculated as a beta (β) value, which is interpreted as the average methylation for a particular site taking into account all cells forming a body fluid sample. Beta values can range from 0, representing the unmethylated sites to 1, corresponding to those completely methylated. Prior to analysis, genome-wide DNA methylation datasets underwent a quality control analysis to account for common experimental biases, such as batch effects using the IBM SPSS v.22 software. Therefore, we used overall mean detected methylation levels to normalise the methylation levels between different datasets, but without removing the occurring DNA methylation variation, partly explained by age. We used the normalised methylation values for age prediction analysis.

### 2.2 Selection of potential age-associated CpG sites

The ability to accurately predict age regardless of the tissue type would be very advantageous in criminal investigations where the identification of the tissue source of a sample is often challenging. Even if the purpose of this study was to identify age-associated CpG sites in blood, the ability to apply a potential model in other tissues with similar accuracy would save both time and resources. In an attempt to select more robust age-associated differentially-methylated markers across tissues, the study by Horvath [48] was chosen as the most appropriate. The author built an age prediction model applicable in various tissues using a total of 353 markers, which were categorised by a coefficient value (ranging from −1.719 to 3.067) that relates the CpG sites to a transformed version of age. In order to cover all potential correlations with age and maximise the chance of selecting suitable markers, 45 CpG sites from the 353 marker pool included in Hovarth’s model were selected, specifically this included those displaying the highest (positive/negative) coefficients (Table S3). Their chromosomal location was confirmed using the Ensembl genome browser; most are located within or near a gene. While it has previously been demonstrated that the ELOVL2 marker can be a good predictor of chronological age in blood [24], there is an absence of genome-wide data for the relevant CpG sites, hence these sites could not be included in this study.

### 2.3 Statistical analysis

Statistical analysis was performed using STATISTICA software v.13.1 (StatSoft Inc., 2014, Oklahome, United States). To assess data distribution, the minimum, maximum, mean and standard deviation (SD) were calculated. Hypothesis testing was evaluated by calculating *p*-values with a significance cut-off of 0.05. Multivariate analysis was used to assess if other defined factors in the datasets (such as sex) were significantly associated with age. The degree of linear association between methylation levels and chronological age was measured by calculating the correlation coefficient (r), while a general regression model, implemented using a forward stepwise approach, was used to assess the accuracy of age prediction with the selected marker candidates. The fitted regression line explains a proportion of the variability in the dependent variable (y) and the residuals indicate the amount of unexplained variability. The proportion of the total variation explained by the model was also assessed by the goodness-of-fit of the line (R^2^ value). In some cases, regression lines were linear but there were cases where the relationship between two variables was curved revealing non-linear relationships. For example, in methylation quantification by bisulfite PCR the polynomial regression was often observed either as quadratic curves (y = ax^2^ + bx + c) or cubic curves (y = ax^3^ + bx^2^ + cx + d).

### 2.4 Artificial neural network (ANN) modelling

The age-specific CpG site methylation data was used to build, train and test a suitable ANN for chronological age prediction. In this study, several ANN types including 2- and 3-layer multi-layer perceptrons (MLPs), radial basis functions (RBFs), probabilistic neural networks (PNNs) and generalised regression neural networks (GRNNs) were built and their performance critically evaluated using Trajan v6 software (Trajan Software Ltd., Lincolnshire, UK). Briefly, optimisation of each ANN type and architecture was performed in a number of stages. Firstly, all input variables (45 selected age-associated CpG sites) were included in the initial design phase to elucidate which ANN model type was likely to be most applicable to age prediction. Following this, the most promising network type (GRNN) was optimised further in a series of stages. GRNNs are a type of ANN that use a combination of a radial basis and linear functions to perform the output estimation [53]. The first optimisation stage was performed to finalise training and verification dataset proportions by assessing the performance of the mean inaccuracy of the blind test sets. Depending on the application, between 50% and 70% of the full 1156 cases were assigned as training cases with equal splitting of the remaining proportion between verification and blind test cases. This was repeated several times for each proportion value and with random assignment of cases for training, verification and testing every time. The network designer tool was set to balance GRNN verification set errors against network diversity to cover as many architectures as possible across all model types. In total, 10^8^ architectures were investigated in each stage and 50 of the best GRNN networks were ranked by correlation and output error separately for the training, verification and blind test datasets. In Stage 2, advanced random sampling was applied whereby training and verification cases were assigned as per the best performing GRNN architecture from Stage 1, but blind test cases were fixed. In Stage 3, the reproducibility of ten replicates of the model was assessed by selecting the best GRNN from Stage 2 and fixing all training, verification and blind test subsets and the best model overall was then selected from this pool. The variability in age estimations for all subsets across all replicate GRNN models was then expressed as mean error ± one standard deviation. Following this, all ten networks were each subjected to a sensitivity analysis to assess the relative contribution of each CpG site input to model accuracy. The error ratio was calculated as the ratio of the observed GRNN test error using all variables to the error obtained when each variable was systematically removed. Larger error ratio values represented more network dependency on that specific CpG site variable. All input variables were also assessed for collinearity using SPSS Statistics v23 (IBM Corporation, New York, USA).

### 2.5 Body fluid samples and DNA preparation

A set of whole blood samples were collected to test the developed ANN model with the proposed methodology. The present study was carried out following full ethical approval by King’s College London Biomedical Sciences, Dentistry, Medicine and Natural & Mathematical Sciences Research Ethics Subcommittee (BDM/13/14-30). Full informed consent was obtained from the donors or their parents in case of under-aged individuals prior to collection. Whole blood was collected from a total of 46 individuals aged 11–76 years old coming from various ethnic backgrounds. Genomic DNA was isolated from 200 μl of whole blood using the BioRobot EZ1 DNA blood kit (QIAGEN, Hilden, Germany). Following purification, samples were quantified using the Quantifiler Human DNA Quantification kit (Applied Biosystems, Foster City, United States). 500 ng of each DNA sample was used for bisulfite conversion using the MethylEdge Bisulfite Conversion system (Promega, Madison, United States) and bisulfite-treated DNA was eluted in 20 μl of elution buffer. For control and linearity analysis, a set of DNA standards of known methylation levels ranging from 0% to 100% (EpigenDx, Hopkinton, United States) were used.

### 2.6 Bisulfite PCRs

In this study the online Ensembl genome browser (GRCh37/hg19) genome was used to obtain the required genetic information for assay design. Primers were designed to specifically amplify bisulphite-treated DNA using the online-tool BiSearch [54] and design parameters were adjusted to account for the generally low efficiency of bisulphite PCR and common mis-priming events due to the T-richness of the bisulphite-treated DNA sequences. A total of 16 singleplex assays were designed to investigate the selected age-associated CpG sites; each bisulfite PCR assay includes a PCR primer set (forward and reverse), none of which binds to areas containing other CpG sites to avoid potential bias (Table 1). Information on the obtained amplicons including their chromosomal location and number of included bisulfite-conversion controls and CpG sites are presented in Table S4. Although the assays were primarily designed to interrogate the 16 selected CpG sites, sequencing of the entire PCR product on the MiSeq^®^ platform (Illumina, San Diego, United States) allowed for the co-analysis of all adjacent CpG sites included in the fragment. Briefly, PCRs were carried out in 13 μl reaction volumes containing a final concentration of 1X ZymoTaq premix (Zymo Research, Irvine, United States), 3.2 mM MgCl_2_, 0.4 μM forward and reverse primers, with the addition of 1 μl of bisulfite DNA template. The thermocycling program used was: 95 °C for 10 min, followed by 30 cycles of 94 °C for 30 s, T_m_ for 30 s, 72 °C for 30 s, and a final extension step of 72 °C for 7 min. The optimised T_m_ was as follows: 48 °C for cg07158339, cg17274064, cg02085507, cg20692569 and cg02479575, 50 °C for cg19761273, cg27544190, cg01511567, cg24450312 and cg04528819 and 52 °C for cg03286783, cg05442902, cg08370996, cg04084157, cg22736354, cg06493994. Following amplification, the quality of PCR products was assessed on a 2% agarose gel if necessary.

### 2.7 Next generation sequencing using illumina MiSeq^®^

Singleplex PCR products were pooled together and purified using the MinElute PCR purification kit (QIAGEN) in 16 μl of DNase-free water. Prior to library preparation, all purified samples were quantified using the Qubit dsDNA HS Assay kit (Invitrogen, Carlsbad, United States) according to the manufacturer’s instructions and in combination with the Qubit 2.0 Fluorometer instrument. Pooled PCR products were diluted appropriately to provide 50 ng of amplified DNA within 25 μl. Library preparation was performed using the KAPA Hyper Prep kit for Illumina (Kapa Biosystems, Wilmington, United States) with half volume reactions. Library amplification proceeded with 8 cycles while the clean-up steps were performed using the AMPure XP Beads (Beckman Coulter Genomics, Danvers, United States) and the Illumina Resuspension buffer (Illumina). To assess libraries’ quantity, purified libraries were diluted 1:4000 in DNase-free water and quantified using the KAPA Library Quantification Kit for Illumina platforms (Kapa Biosystems). Indexed DNA libraries were then normalised to 4 nM using Tris-HCL 10 mM/pH 8.5 with 0.1% Tween and were pooled together to a final volume of 240 μl. Using freshly made 0.2N NaOH, 5 μl of pooled libraries were denatured and, through dilution with pre-chilled Hybridisation buffer (HT1, Illumina), a 10pM library was obtained. Finally, 13% diluted PhiX control (80 μl) was added to the library and sequencing was performed using the 300-cycle MiSeq^®^ reagent v2 cartridge (Illumina). The preparation of the flow cell and the set-up of the instrument were performed per manufacturer’s instructions. It should be noted that the instrument was set up to run a paired-end read of 150 bp of DNA sequence from both ends of the library products. Auto analysis was set up as a FASTQ-only method.

Following auto-analysis by the MiSeq^®^ Reporter Software, which separated the millions of generated sequences into the constituent samples on the basis of the ligated adaptor tags, collated sequences were packaged in a text-based format (FASTQ files). For alignment, we used a custom bisulfite-converted reference genome containing all analysed DNA sequences, which is quicker and more user-friendly compared to available alignments using the entire genome. Sequences within these FASTQ files were aligned using a Burrows-Wheeler alignment (BWA) algorithm. This process was implemented in the BWA program [55] using the maximum entropy method (mem) algorithm that matched the sequences generated to the respective methylation marker (i.e. a sequence obtained from the PCR product of any specific marker would be most similar to the reference sequence for that marker, and hence the software would align this sequence with that marker). Therefore, the millions of sequences contained within the FASTQ file can be associated with their respective marker, giving potentially hundreds of thousands of individual sequences all aligned in parallel to a specific reference sequence/marker. At the conclusion of this alignment process, a sequence alignment/map (SAM) file was produced, which was further modified using SAMtools [56] to facilitate the conversion into a BAM file. The Genome Analysis Toolkit (GATK) [57] was subsequently used to interrogate this BAM file by targeting specific positions in these aligned sequences (i.e. each CpG site) and reporting the number of sequences containing a C and the number of sequences containing a T at this position. In this way it was possible to assess the methylation state at every studied CpG site. The unified genotyper algorithm was employed within GATK to produce these genotype data for each CpG site, which were written into a variant call format (vcf) file that could subsequently be manipulated in Excel. The Integrative Genomics Viewer software (IGV) was used for visualisation and verification of the alignment. While in most cases the bisulfite conversion rates were >99%, methylation values were ‘corrected’ by taking into account the mean bisulfite conversion rates per fragment calculated by the built-in conversion controls (non-CpG cytosines). The obtained methylation values for each CpG site were further normalised using the resulting equations of standard curves created from known DNA methylation standards. Lastly, to account for potential methodology-dependent differences, the NGS derived methylation values were normalised to the genome-wide data, used to build the prediction model, by applying the method previously described when normalising the different genome-wide data sets.

## 3 Results and discussion

### 3.1 Age-associated DNA methylation changes in blood

**Fig. 1:**
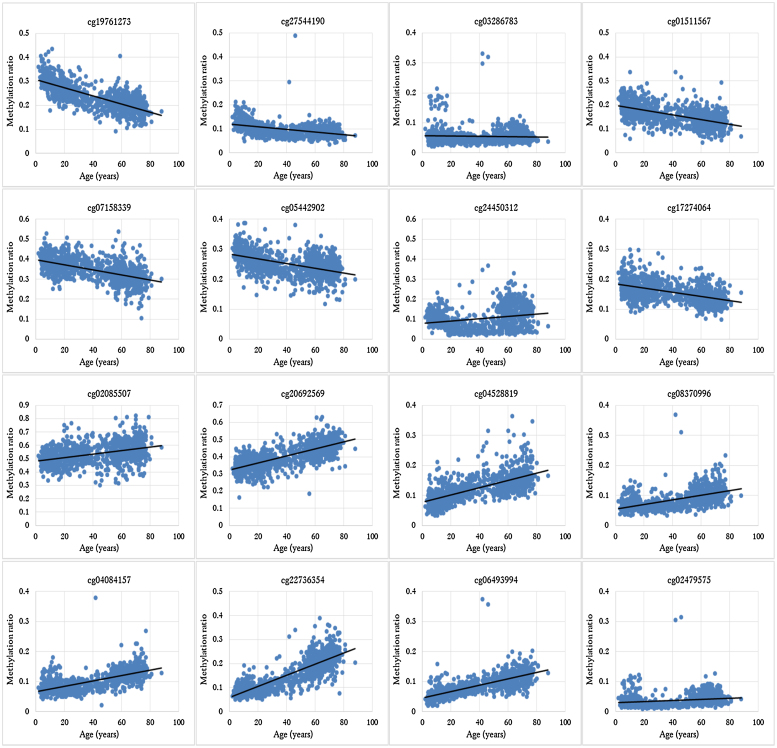
Change of methylation levels over advancing age for the 16 CpG sites included in the eventual ANN model.

Using publicly available DNA methylation databases, normalised beta values for the selected 45 CpG sites were gathered for a total of 1156 whole blood samples from individuals 2–90 years old. Methylation fractions (zero to one) were compared against the actual age of each individual in order to investigate potential correlation between methylation levels and age (example graphs for 16 out of 45 CpGs are presented in Fig. 1). As expected, some CpG sites showed greater variation than others; for example, cg07455279 (NDUFA3) demonstrated the largest methylation range (difference between the lowest and highest detected methylation value for each marker) (0.815) while cg05442902 (P2RXL1) usually showed low methylation levels (<0.387). In general, the methylation of certain CpG sites such as cg19761273 (CSNK1D), cg01511567 (SSRP1), cg07158339 (FXN) and cg05442902 (P2RXL1) was clearly decreasing with advancing age, while others, cg20692569 (FZD9), cg04528819 (KLF14), cg04084157 (VGF) and cg22736354 (NHLRC1) to name but a few, were increasingly methylated over time. These observations align with the age relationship that Horvath reported in his study [48].

### 3.2 Identification of the epigenetic aging signature

The observed age-associated methylation changes for all markers were assessed for their statistical significance in an attempt to identify those sites that could form the proposed epigenetic-aging-signature. Firstly, using multivariate analysis and testing for the effect of gender and ethnicity on age-associated methylation, no significant correlation was determined (p = 0.77 and p = 0.09 respectively). Using linear regression analysis, a significant correlation between methylation levels and age (p < 0.05) was confirmed for 25 out of the 45 CpG sites (Table S5). Applying stepwise multiple linear regression for variable selection, we obtained similar results regarding the importance and order of markers (Table S6). In order to perform this type of analysis, the markers were added one by one into the age prediction model until there was no statistical improvement. As a result, the use of 23 CpG sites resulted in a value of R^2^ = 0.923, which was not further improved with the addition of more markers. All 23 age-associated CpG sites revealed following stepwise regression are included in the set of markers identified after individual linear regression analysis. In both analyses cg22736354 (NHLRC1) was found to be the most important. Interestingly, while cg07455279 (NDUFA3) had demonstrated the higher methylation range, it did not demonstrate a statistically significant correlation with age, which mirrors the complexity and high, inter-individually variable nature of methylation patterns.

**Fig. 2:**
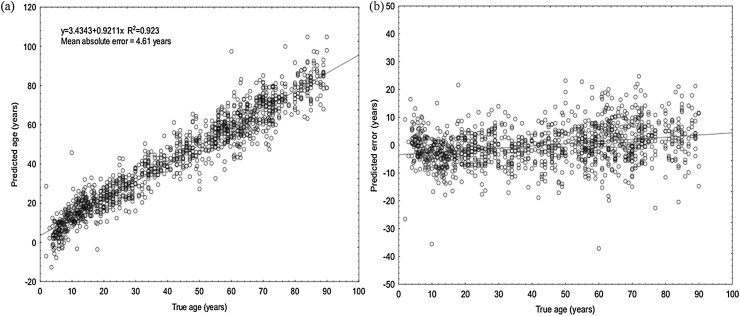
Age prediction using multiple regression analysis (23 CpG sites) (a) Predicted *vs.* Chronological age (years) for all 1156 individuals used in this study (linear correlation R^2^ = 0.923, mean absolute error = 4.61 years, standard deviation = 4.36 years), (b) Predicted error (years) over advancing age. As shown most individuals were predicted within a ±5 year error range (0.61), while 1029 out of 1156 samples were predicted within a ±10 year error range (0.89).

Applying multiple linear regression analysis on the methylation values of all 1156 individuals for the 23 age-associated CpG sites, the correlation between fitted and true age was strong (linear correlation, R^2^ = 0.923), while the mean absolute age modelling error using all data was 4.61 years (standard deviation = 4.36 years) (Fig. 2a). In a brief summary, 61% (700/1156) of individuals were fitted within a ±5 year error range, while 89% (1029/1156) of samples were fitted within a ±10 year error range. Multivariate regression was mainly applied to explore the relationship between DNA methylation patterns and age, but also revealed that none of the available factor variables (gender, ethnicity) influenced age-associated DNA methylation patterns in a statistically significant way. While there were individuals that seemed to age fast, there were also others that were fitted as much younger (Fig. 2b). Notably, the error in older individuals (>60 years old) was higher compared to younger ones, which is not only expected as older individuals have been exposed to more environmental stress throughout their lifetime that could have potentially caused changes in DNA methylation patterns (epigenetic drift), but has also been observed before in previous models [25], [33].

### 3.3 Age predictions from blood using artificial neural networks

**Fig. 3:**
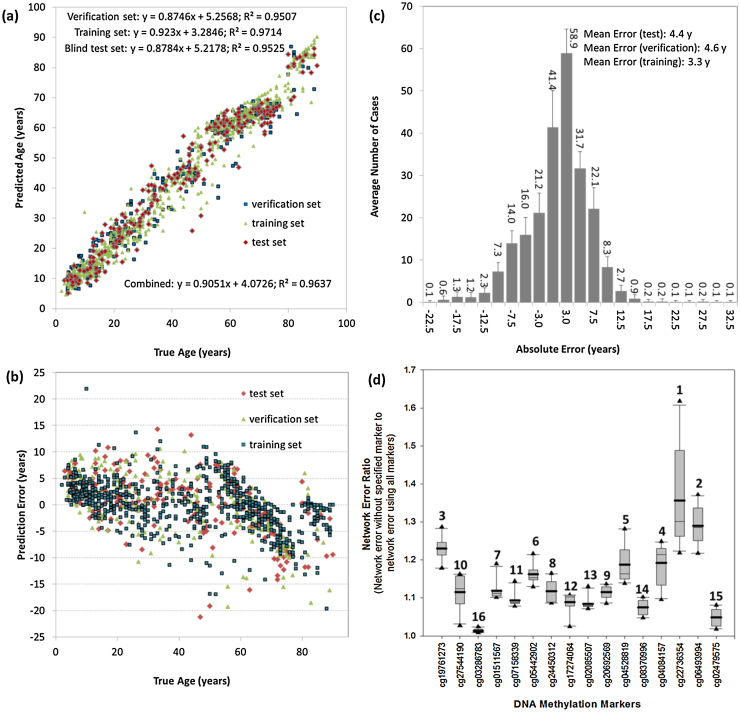
Summary of ANN model for age prediction analysis. (a) Predicted *vs.* Chronological age for all 1156 individuals included in the study using the optimised 16–694-2-1 GRNN model, (b) Residual errors for the optimised model, (c) Prediction skewness for the blind test cases only using the optimised model, and (d) Sensitivity analysis and marker input consistency to age predictions across training, verification and blind test subsets. Error ratios are calculated as the ratio of the prediction inaccuracy by including all inputs to the prediction accuracy following systematic removal of each CpG site from 10 replicated GRNN networks. Boxes include data from the 25th–75th percentile as well as the median (thin line) and mean (thick line); error bars include the 5th and 95th percentile; numbers over boxes represent the rank order based on the mean.

Neural network models showed that the prediction accuracy could be significantly improved over multiple linear regression models. It is believed that ANN models have the ability to recognise complex patterns, which are often observed in complex traits like chronological age. The best model (Fig. 3a) was a 16-694-2-1 GRNN-type model, which was built on a 60:20:20 training, verification and blind test set dataset proportion (optimised). The average absolute errors and standard deviations in each of these subsets were 3.3 ± 3.0, 4.6 ± 3.5, and 4.4 ± 3.6 years, respectively (Fig. 3b). As a whole, a correlation between predicted and true age of R^2^ > 0.96 was achieved across all subsets with an average absolute error of 3.8 ± 3.3 years. The correlation for the blind test set (R^2^ = 0.95) was consistent with both the training and verification sets showing that the model could generalise very well. For the blind test set in particular, the 75th percentile of all 231 case errors lay within 6.3 years. This performance is consistent with other ANN-based applications from our research group which revealed a 3–5% average inaccuracy across predictions [58] and with a recent study reporting a percentage of prediction error of 6.3% [38].

This ANN model used data from 16 of the CpG sites, all 16 of these sites having also been identified in the stepwise multiple regression analysis: the methylation changes over time for these 16 markers are illustrated in Fig. 1. While a distinct methylation trend is observed in all cases, there are occasional samples that demonstrate an ‘unusual’ methylation status for a few CpG sites. This can be explained either as natural inter-individual variation or as a result of a ‘unique’/personalised environment that could influence the methylation of these particular sites. Of course, technical variation cannot be excluded, however efforts were made to take this into account and normalise the data before analysis. Information regarding the genes that the CpG sites lay near or within was acquired to identify their function and potential involvement in aging. The exact chromosomal locations of the CpG sites as well as the involved genes are shown in Table 2.

As expected, all markers showing significant correlation with age belong to genes involved in age-related processes and conditions; a few examples are presented here. cg19761273 is associated with CSNK1D, which is a serine-threonine protein kinase involved in essential cell pathways including circadian rhythms and DNA repair. It is believed that CSNK1D has a role in arranging the microtubule network during mitosis to prevent DNA damage [59]. On the same theme, SSRP1 linked with cg01511567 seems to be crucial to anticancer mechanisms since it forms a transcriptional factor that interacts specifically with histones and prevents DNA damage [60]. Additionally, cg03286783 belongs to the *CASC4* gene, increased expression levels of which have been found in breast and ovarian cancers [61]. Moreover, cg05442902 is associated with the *P2RXL1* gene known for its involvement in inflammatory and immune processes, all affected by aging [62]. A recent study has also linked *TRIP10* gene (cg02085507) with the regulation of cancer cell growth; in fact differential DNA methylation of this gene has been suggested to promote cell survival or death [63]. Additionally, cg04528819 belongs to transcription factor KLF14, which is known as the ‘master regulator’ of obesity and other metabolic traits [64]. Lastly, the nerve growth factor VGF associated with cg04084157 has been linked with altered expression levels in the age-associated Alzheimer’s disease [65]. Even though some of the genes are associated with age-related diseases, Fig. 1 demonstrates a gradual and consistent methylation increase or decrease over time at these particular CpG sites; therefore, their detected correlation with age should not be linked with effects due to these individuals being affected by age-related conditions.

The residual error obtained by the GRNN (Fig. 3b) displayed a distinct, imbalanced pattern in comparison to that obtained from multiple regression analysis (Fig. 2b), which was more randomly distributed around the mean. Residual errors obtained by the GRNN for old individuals (>60 years old) revealed a different grouping within the graph as the model tends to underestimate their age. These findings could indicate that there is a potential ANN bias in age prediction within specific age groups, that could perhaps be corrected in the future, or that there is a learning artefact due to so many different study datasets being combined. Lastly, the skewness of the blind test predictions alone showed that there was a marginal bias towards under-prediction of age using the GRNN model (Fig. 3c). In an attempt to further understand the relative contribution and consistency of each CpG site variable for age prediction, ten replicate GRNNs were generated. Sensitivity analysis showed that all error ratios lay above 1.0 meaning that they all contributed to the model positively (Fig. 3d). Most notably, cg22736354 (NHLRC1) and cg06493994 (SCGN) contributed the most to prediction, which was also true for the former in the stepwise regression analysis. Some moderate collinearity existed between these two variables and so the relative ranking of either should be considered carefully (variable inflation factors were 6.172 and 7.743 respectively, see Table S7 & S8), which has also been observed in previous models [38]. Overall, it was decided not to remove either variable from the network as predictions became worse overall, even after re-training. From the third highest ranked variable (cg19761273, CSNK1D) onwards, no severe collinearity existed and therefore these rankings were more reliable. Despite contributing to the prediction in a minor way (error ratio = 1.0127), cg03286783 (CASC4) was the lowest ranked of all 16 variables across all replicate GRNNs. Consistency of contribution across all ten GRNNs was also acceptable and in general error ratios varied <0.1 units for 25th–75th percentile of all data.

Considering that a similar prediction accuracy was observed when using 353 CpG sites in Horvath’s study (age correlation of 0.96 with a median absolute error of 3.6 years), predicting age with high accuracy using a smaller number of CpG sites (16 in our case) was possible (mean absolute error of 4.4 years in the blind test set). This is also supported by previous studies where researchers obtained mean prediction errors of 4–8 years in their validation tests, such as 5.1 years using 8 markers by Zubakov et al. [39], 6.9 years using 3 markers by Park et al. [30], 4.2 years using 7 markers by Freire-Aradas et al. [38], 3.9 years using 5 markers by Zbieć-Piekarska et al. [25], to name but a few. To the best of our knowledge, only one of the proposed 16 age-associated genes in our study has been used before in a forensic age model (more specifically, KLF14 is included in the Zbieć-Piekarska model), therefore contributing towards building a bank of potential markers. As shown before, our model sensitivity analysis revealed that there were markers contributing more to age prediction, therefore, one could propose that by replacing or adding some of the other ‘strong’, age-associated CpG sites reported in the literature, such as the example of *ELOVL2* locus [24], the resulting prediction accuracy can be further improved. Also, for future studies, one should also consider the combination of the best age-associated CpG markers with other age-related molecules, like mRNA, as this can also improve accuracy [39].

### 3.4 Validation through an independent cohort of monozygotic twins

Even though the model was applied to 231 blind test cases in the model optimisation stage, it was important to externally test model performance with an independent cohort of samples. For this reason, we evaluated the optimised model using 106 blood samples belonging to 53 monozygotic twin pairs aged 33–77 years. Monozygotic twins were chosen since they begin life with nearly identical genetic and epigenetic profile and it is the effect of various environmental factors that alters their genome-wide DNA methylation profile later in life. The methylation values of each sample for all 16 CpG sites were imported into the model as a blind test and the average mean absolute error was 7.07 ± 5.78 years. This higher prediction error could be partly explained by the fact that most twins were old (mean age of 58 years in this dataset), therefore the effect of environmental conditions and lifestyle should be considered. Interestingly, between pairs there were twins that were predicted to be either much older or much younger than their actual age, but the prediction differences within twin pairs (mean = 2.65 ± 2.37 years) were not statistically significant as obtained by paired *t*-test analysis (*p*-value = 0.99). These results can be interesting since they indicate some sort of systematic influence of either the twins’ genetics or environment. According to Horvath, while the heritability of age acceleration was found to be 100% in new-borns, it was only 39% in older subjects suggesting that non-genetic factors become more relevant later in life [48]. Also, although all twins were volunteered as healthy controls, it would be beneficial if information regarding disease status or susceptibility was available, that could possibly partly explain these results.

### 3.5 Effect of disease state on age predictions

It is important to bear in mind that, in contrast with a medical setting, information regarding possible disease status is not available when trying to predict chronological age from an unknown bloodstain or sample during a criminal investigation. Consequently, it is important to build a robust age prediction model containing DNA methylation markers that would not show differential methylation patterns due to disease states. However, this might be extremely challenging to do. Therefore, although Horvath has already reported that the predicted age from cancer tissues correlated poorly with patient age in his study [48], we aimed to investigate a set of diseased samples and the effect on age prediction. For this purpose, seven datasets including diseased samples were analysed in an attempt to further validate the proposed age prediction model (Table S2). Fig. S2 shows the predicted *vs.* chronological age for all 1011 samples; combining all diseases together, a correlation of 0.74 and a mean absolute error of 7.18 years was obtained. However, when analysing separately samples suffering from blood *vs.* non-blood related diseases it becomes evident that the error is much higher for blood related diseases (error = 12.74 years). This is of course expected since the methylation data were gathered by analysing whole blood samples and therefore the potential effect is direct. In more detail, the obtained mean absolute errors for each disease were as follows: type I diabetes – 8.63 years, anaemia – 14.38 years, bone marrow disorders (including leukaemia) – 11.09 years, ovarian cancer – 7.45 years, breast cancer – 6.77 years and schizophrenia – 5.03 years.

Schizophrenia showed the lowest age prediction error, while anaemia demonstrated the lower correlation with age. While changes in expression of one of the markers included in the model – cg04084157 (VGF) – have been detected in the cerebrospinal fluid of patients with different neurological and psychiatric conditions such as schizophrenia [66], it did not seem to affect prediction in blood. It should also be noted that schizophrenia patients comprised the largest dataset; therefore a better prediction error could also be due to the greater number of samples. On the other hand, the results regarding anaemia (n = 28) come as no surprise since anaemia is one of the most common blood disorders, which could add extra ‘stress’ on the body and alter DNA methylation patterns, especially in blood. Interestingly, cg07158339 is located near the *FXN* gene which has been associated with selectively and non-covalently interacting with ferric ion Fe (III) to assemble the iron-sulphur cluster [67]. Consequently, differential methylation patterns due to the disease status in blood cannot be excluded. Another example includes the *ERG* oncogene associated with cg17274064, which is an erythroblast transformation-specific transcription regulator typically mutated in myeloid leukaemia [68]. As shown, the dataset comprised by various bone marrow disorders including leukaemia demonstrated the second largest mean error (11.09 years). Thus, by testing this limited range of diseases, it seems that our model has the potential to perform quite well in disease-stressed samples unless these are blood-related, and the possibility of disease state should be taken into account when attempting predictions.

### 3.6 Applying the age prediction model in saliva

In Horvath’s study, this set of 353 markers, which included our 16 CpG sites, were used to predict age in a wide range of other tissues. However, individual marker capabilities should not be overlooked, therefore to assess potential tissue-specific variations in age prediction, the selected markers were also tested in a set of saliva samples. Saliva is not only one of the most common types of biological evidence found at crime scenes in the form of used glasses, cigarette butts or stamps, but also was the only tissue where sufficient genome-wide methylation data was available for robust analysis. One confounding factor when analysing saliva methylation data is the variation derived from the collection method used. Depending on the method applied to collect saliva (for example by mouth wash, oral fluid swab or by ‘touch’ samples), the body fluid stain might contain differing proportions of various cell types (for example, buccal epithelial cells and white blood cells), which can result in detecting variable DNA methylation levels. Methylation values regarding the selected 16 CpG sites were collected from a total of 265 samples of individuals aged 21–55 years; while 159 samples were used to train a GRNN model, 53 samples were used for each of the verification and blind test sets. As shown in Fig. S3, a good correlation of 0.73 was obtained between predicted and true age, while the mean error was 3.18 years (training), 6.26 years (verification) and 4 years (blind test). The prediction accuracy was encouraging considering the size of the dataset; however, the narrow age range (21–55 years) cannot be ignored. Furthermore, saliva was collected and extracted differently between the two studies used, which could introduce further variation. The majority of saliva samples used here were collected via the Oragene DNA collection kit, which can typically result in DNA being extracted primarily from white blood cells, rather than buccal epithelial cells; which, in this case, could explain the high accuracy of obtained predictions. Again, as shown in the graph representing the age residuals, age prediction seemed to be more accurate in younger individuals, where underestimating age was not very common. Even though including more saliva-specific age-associated CpG sites could significantly improve the obtained prediction error, these results highlight the potential applicability of the proposed model in non-blood tissues.

### 3.7 Model validation by means of next generation sequencing

**Fig. 4:**
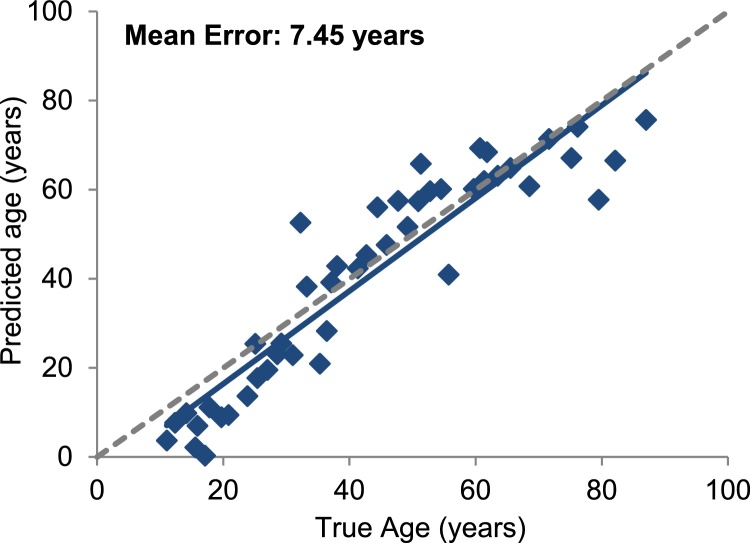
Age prediction in blood using the developed MiSeq method (n = 46).

Our last goal in this study was to implement our age prediction model by using an accurate, robust and sensitive method that can analyse the proposed CpG sites in forensic-type samples. Compared to previous analog methylation methods used for age prediction analysis, we strongly believe that NGS can show great potential, as not only it can be more sensitive and accurate, but can also provide data of higher-resolution. Therefore, an NGS-based protocol capable to detect DNA methylation differences in bisulfite-converted DNA fragments was developed and adjusted using a previously published method [69]. The overall performance of the method was good including <0.05 standard deviation in methylation detection for most markers, even though we observed an imbalance between the average reads of the investigated fragments; specifically, cg24450312 (RASSF5) and cg17274064 (ERG) were the most challenging markers. This could be due to different PCR efficiencies explained by DNA sequence differences among markers, and can be improved in future experiments. Nevertheless, to ensure accurate methylation quantification, a minimum of 1000 reads per marker was set. For prediction analysis, a set of 46 blood samples from individuals aged 11–76 years old were analysed in triplicate using the proposed method and their predicted age was calculated using the average as a blind test in our age model. As a result, the age correlation taking the final normalised methylation data was 0.86 and we could predict age with a mean absolute error of 7.45 years (Fig. 4).

These results are very encouraging, and even though the age prediction accuracy is lower than that obtained in the model’s blind test, for this sample set we are introducing an additional layer of variation when normalising methylation values between totally different detection systems (the NGS-derived methylation values in the samples with the microarray-derived ones used to train the model). As we aimed to investigate a representative population sample, no information regarding the individuals’ health and lifestyle were collected as this information would not be available to a ‘standard’ forensic scenario. Since DNA methylation is known to not only be age-specific but can also be influenced by diet [70], lifestyle [71], smoking [14], ancestry [72] and other factors, we cannot exclude that these factors could have affected the methylation status of the selected sites in these individuals. However, even if efforts were made to normalise the NGS data over the genome-wide methylation data that the age prediction model uses, we cannot also ignore other potential (PCR-introduced) technical variation. Current experiments focus not only on increasing the sample size to achieve a more representative prediction accuracy, but also to analyse enough samples to re-train the model with NGS data; the latter would eliminate any methodological or technical variation. Furthermore, we are also investigating the possibility of multiplexing the bisulfite PCRs to allow for more sensitive analysis and validating the entire method using forensically relevant criteria. We understand that both the required high coverage (1000X) and triplicate analysis may be impractical in routine forensic analysis, albeit less so for blood traces where DNA is often, although not exclusively, found in relatively high quantities. Future efforts should therefore also be concentrated on extensively testing the sensitivity and reproducibility of the proposed NGS based method. We believe that introducing an NGS-based solution for age prediction can provide many advantages from a casework point of view, mainly due to its high sensitivity, multiplexing capabilities and the potential for merging with other DNA marker analysis. Nevertheless, all of the factors mentioned above, including the biological variation that may result from disease state and lifestyle, need to be established before such methods can be applied routinely in forensic casework.

## 4 Conclusions

Forensic age prediction using DNA methylation-based approaches is a fast-developing field of forensic epigenetics that has a great potential to provide accurate outcomes. Our study contributes to a range of already published prediction models, not only by providing potential age-associated markers but also by introducing a novel methodology in prediction analysis, namely machine learning by artificial neural network analysis. The proposed age prediction model does not only exhibit good prediction accuracy, but also has the potential to be applied in individuals of a very wide age range including under-aged children, individuals of various ethnic backgrounds as well as in non-blood tissues. Nevertheless, it is believed that prediction can be improved in the future by normalising for the different technologies of DNA methylation analysis used. Also, the model worked significantly less accurately in a subset of unhealthy individuals, therefore testing the markers’ ‘resistance’ to DNA methylation alterations in disease state should be further tested. To the best of our knowledge, this is the first study that tests the ability of next generation sequencing technology to detect DNA methylation variation for age prediction in forensic samples. Following an extensive validation in future experiments it could provide the basis to an eventually combined analysis of DNA methylation and DNA sequence variation in a single streamline using an NGS platform.

## Conflicts of interest

Authors declare no conflict of interest.

## Figures and Tables

**Table 1:** Designed bisulfite PCR assays.

CpG site	Gene	Primer Sequence (5′-3′)		Amplicon Length (bp)
cg19761273	CSNK1D	F	TGTTTAGTTTGAAGATTGAG	150
		R	CCTTATTTCCTTTACAAAAA	
cg27544190	C21orf63	F	GGGTAGGATTAAAGTTGA	106
		R	CTTAAAAATAACAATCCCC	
cg03286783	CASC4	F	GTTTTAGTTAGTGGGTG	181
		R	CCCCTCCTCAAATCAAA	
cg01511567	SSRP1	F	TATTAGATTTAGTATAGGGG	132
		R	CCCACAACTATTCAAATA	
cg07158339	FXN	F	GGAATATGTTTTGTTTAAAA	122
		R	TAATTAACCTCTCTATACCT	
cg05442902	P2RXL1	F	GTATGTTTTGGTTTTTGT	109
		R	AATAACCTCTAAACTAACC	
cg24450312	RASSF5	F	GTTATTTATAGAGTTTGAG	201
		R	TCTACTACAAACCAAA	
cg17274064	ERG	F	AGGGAATAAGTATTTTTT	139
		R	CTCACAATCAAACTTCTATATAC	
cg02085507	TRIP10	F	GTTAATGGATTTGGTTTTG	186
		R	AACTCAAAAAATCCTTCCT	
cg20692569	FZD9	F	TTGTTGTTGTGGTAGT	160
		R	AACCCAACAAATTAAA	
cg04528819	KLF14	F	AATAGGTTTTGGTGTAGTT	138
		R	CAACCTCTAATAAATTCTCT	
cg08370996	NR2F2	F	GTGTTAAAGTTTATTATATAGA	187
		R	AAAAAAAAAAACACACAC	
cg04084157	VGF	F	GAGGGTGTTTGTTTTTTT	111
		R	AACATTTCATTCATTCATTC	
cg22736354	NHLRC1	F	GTTGAGTTTAGGAGTTTTAT	201
		R	CTTTAAAAAATTTAACCACC	
cg06493994	SCGN	F	GGAGAGTAAGTTAAGAAATA	150
		R	AACCTACCAAAAACCAAC	
cg02479575	C19orf30	F	GGAGGAGAATGTTATTTATT	143
		R	CTATCCAAAATTCTAAAAAC	

**Table 2:** Epigenetic aging signature consisted of 16 CpG sites Information in this table includes the exact chromosomal location of the selected CpG sites (GRCh37/hg19) as well as the involved genes.

CpG sites	Chromosomal location	Gene
cg19761273	17: 80,232,096	CSNK1D − casein kinase 1; delta isoform 1
cg27544190	21: 33,785,434	C21orf63 − chromosome 21 open reading frame 63
cg03286783	15: 44,580,973	CASC4 − cancer susceptibility candidate 4 isoform a
cg01511567	11: 57,103,631	SSRP1 − structure specific recognition protein 1
cg07158339	9: 71,650,237	FXN −frataxin, mitochondrial isoform 1 preproprotein
cg05442902	22: 21,369,010	P2RXL1 − purinergic receptor P2X-like 1; orphan receptor
cg24450312	1: 206,681,158	RASSF5 − Ras association domain family 5 isoform B
cg17274064	21: 40,033,892	ERG − v-ets erythroblastosis virus E26 oncogene like isoform 2
cg02085507	19: 6,739,192	TRIP10 − thyroid hormone receptor interactor 10
cg20692569	7: 72,848,481	FZD9 − frizzled 9
cg04528819	7: 130,418,315	KLF14 − Kruppel-like factor 14
cg08370996	15: 96,874,031	NR2F2 − nuclear receptor subfamily 2; group F; member 2
cg04084157	7: 100,809,049	VGF − nerve growth factor inducible precursor
cg22736354	6: 18,122,719	NHLRC1 − malin
cg06493994	6: 25,652,602	SCGN − secretagogin precursor
cg02479575	19: 4,769,653	C19orf30 − hypothetical protein LOC284424
