# Supplementary material for: DNA methylation-based forensic age prediction using artificial neural networks and next generation sequencing
Source: Forensic Sci Int Genet. 2017 May;28:225–36. doi: 10.1016/j.fsigen.2017.02.009 (PMC5392537; doi:10.1016/j.fsigen.2017.02.009)
Supplement: Supplementary file 1 [file mmc1.docx]

**Supplementary Material**

**Supplementary Tables:**

**Table S1. DNA methylation datasets from various healthy tissues**

The table provides an overview of the selected DNA methylation data used in this study. Information regarding the sample size, gender (F/M), age range, mean age and employed Illumina platform are included for each dataset.

| Tissue | Set | Samples | ♀/♂ | Age range (mean) (years) | Platform | Accession No | Reference |
| --- | --- | --- | --- | --- | --- | --- | --- |
| Whole blood | 1 | 235 | 0/235 | 4-18 (10) | 27K | GSE27097 | [12] |
|  | 2 | 24 | 12/12 | 2-35 (14) | 27K | GSE23638 | [44] |
|  | 3 | 170 | 77/93 | 32-90 (65) | 450K | GSE40279 | [21] |
|  | 4 | 385 | 187/198 | 16-88 (40) | 27K | GSE41037 | [17] |
|  | 5 | 33 | 12/21 | 18-65 (29) | 450K | GSE41169 | [17] |
|  | 6 | 91 | 91/0 | 49-74 (63) | 27K | GSE20236 | [16] |
|  | 7 | 218 | 218/0 | 52-78 (65) | 27K | GSE19711 | [19] |
|  | 8 | 40 | 40/0 | 47-77 (62) | 450K | GSE53128 | [45] |
|  | 9 | 66 | 66/0 | 33-68 (56) | 27K | GSE58045 | [7] |
| Saliva | 10 | 69 | 0/69 | 21-55 (35) | 27K | GSE28746 | [46] |
|  | 11 | 196 | 54/142 | 21-55 (32) | 27K | GSE34035 | [47] |

**Table S2. DNA methylation datasets with various diseases**

The table provides an overview of the selected DNA methylation data used in this study. Information regarding the sample size, gender (F/M) and age range are included for each dataset. All data are publicly available and can be assessed using the Accession No in the Gene Expression Omnibus (GEO) database.

| Disease | Samples (♀/♂) | Age range (years) | Accession No | Study |
| --- | --- | --- | --- | --- |
| Type 1 diabetes | 194 (98/96) | 24-74 | GSE20067 | [49] |
| Anaemia | 28 (24/4) | 23-85 | GSE49904 | [8] |
| Schizophrenia | 324 (81/243) | 17-86 | GSE41037 | [17] |
|  | 61 (16/45) | 18-65 | GSE41169 |  |
| Bone marrow disorders | 77 (31/46) | 28-90 | GSE42042 | [50] |
| Ovarian cancer | 262 (262/0) | 49-91 | GSE19711 | [19] |
| Breast cancer | 30 (30/0) | 50-70 | GSE32396 | [51] |
|  | 35 (35/0) | 24-70 | GSE57285 | [52] |

**Table S3. Selected age-associated CpG sites from the Horvath study**

The table provides information regarding the chosen CpGs including their previously reported age relationship (positive, methylation increasing over time *vs.* negative, methylation decreasing over time) and associated gene.

| **CpG sites** | **Age Relationship** | **Gene** |
| --- | --- | --- |
| cg16408394 | negative | RXRA |
| cg25683012 | positive | BAZ2A |
| cg19761273 | negative | CSNK1D |
| cg27544190 | negative | C21orf63 |
| cg03588357 | positive | GPR68 |
| cg03286783 | negative | CASC4 |
| cg19273182 | positive | PAPOLG |
| cg15703512 | negative | MGC50721 |
| cg01511567 | negative | SSRP1 |
| cg09441152 | negative | PQLC1 |
| cg02047577 | negative | UCKL1 |
| cg17338403 | negative | SLCO3A1 |
| cg07158339 | negative | FXN |
| cg01873645 | positive | C9orf85 |
| cg05442902 | negative | P2RXL1 |
| cg04999691 | negative | C7orf29 |
| cg24450312 | positive | RASSF5 |
| cg04452713 | negative | DST |
| cg22613010 | negative | CLCN2 |
| cg09646392 | negative | TNFSF13B |
| cg17274064 | negative | ERG |
| cg16984944 | negative | TBC1D23 |
| cg00436603 | negative | CYP2E1 |
| cg24126851 | positive | DCHS1 |
| cg14723032 | positive | PITPNM3 |
| cg06926735 | negative | UBE2V1 |
| cg14308452 | positive | MGC24975 |
| cg00374717 | positive | ARSG |
| cg07455279 | positive | NDUFA3 |
| cg02085507 | positive | TRIP10 |
| cg20692569 | positive | FZD9 |
| cg04528819 | positive | KLF14 |
| cg08370996 | positive | NR2F2 |
| cg26297688 | positive | C12orf23 |
| cg23092072 | negative | AFF1 |
| cg04084157 | positive | VGF |
| cg01968178 | positive | REEP1 |
| cg25505610 | positive | hfl-B5 |
| cg06993413 | positive | DPP8 |
| cg00864867 | positive | PAWR |
| cg22736354 | positive | NHLRC1 |
| cg06493994 | positive | SCGN |
| cg02479575 | positive | C19orf30 |
| cg16241714 | positive | CEBPD |
| cg14424579 | positive | FLJ21839 |

**Table S4. Chromosomal locations of DNA methylation assays (GRCh37/hg19)**

| **Assay** | | **Conversion controls** | **CpG sites** | **Chromosomal location**  **to be sequenced** |
| --- | --- | --- | --- | --- |
| **1** | **cg19761273** | 42 | 2 | 17: 80,232,017-80,232,166 |
|  |  |  |  |  |
| **2** | **cg27544190** | 18 | 4 | 21: 33,785,414-33.785.519 |
|  |  |  |  |  |
| **3** | **cg03286783** | 40 | 10 | 15: 44,580,864-44,581,044 |
|  |  |  |  |  |
| **4** | **cg01511567** | 12 | 3 | 11: 57,103,582-57,103,713 |
|  |  |  |  |  |
| **5** | **cg07158339** | 23 | 1 | 9: 71,650,150-71,650,271 |
|  |  |  |  |  |
| **6** | **cg05442902** | 32 | 2 | 22: 21,368,989-21,369,097 |
|  |  |  |  |  |
| **7** | **cg24450312** | 66 | 27 | 1: 206,681,003-206,681,203 |
|  |  |  |  |  |
| **8** | **cg17274064** | 23 | 2 | 21: 40,033,806-40,033,944 |
|  |  |  |  |  |
| **9** | **cg02085507** | 38 | 12 | 19: 6,739,164-6,739,349 |
|  |  |  |  |  |
| **10** | **cg20692569** | 37 | 20 | 7: 72,848,365-72,848,524 |
|  |  |  |  |  |
| **11** | **cg04528819** | 35 | 8 | 7: 130,418,073-130,418,210 |
|  |  |  |  |  |
| **12** | **cg08370996** | 41 | 13 | 15: 96,873,886-96,874,072 |
|  |  |  |  |  |
| **13** | **cg04084157** | 19 | 8 | 7: 100,808,988-100,809,098 |
|  |  |  |  |  |
| **14** | **cg22736354** | 47 | 18 | 6: 18,122,551-18,122,751 |
|  |  |  |  |  |
| **15** | **cg06493994** | 33 | 11 | 6: 25,652,542-25,652,691 |
|  |  |  |  |  |
| **16** | **cg02479575** | 31 | 10 | 19: 4,769,688-4,769,830 |
|  |  |  |  |  |

**Table S5. Correlation of selected CpG sites with age as assessed by their p values (linear regression analysis)**

| **CpG sites** | **p value** |
| --- | --- |
| **cg22736354** | 0.0000001 |
| **cg06493994** | 0.0000001 |
| **cg19761273** | 0.0000001 |
| **cg04528819** | 0.0000001 |
| **cg04084157** | 0.0000001 |
| **cg20692569** | 0.0000001 |
| **cg02085507** | 0.0000001 |
| **cg01511567** | 0.000002 |
| **cg27544190** | 0.000006 |
| **cg05442902** | 0.000044 |
| **cg17274064** | 0.000186 |
| **cg14308452** | 0.000265 |
| **cg16408394** | 0.000601 |
| **cg07158339** | 0.001582 |
| **cg02479575** | 0.002700 |
| **cg23092072** | 0.010637 |
| **cg08370996** | 0.018122 |
| **cg04999691** | 0.02183 |
| **cg24126851** | 0.026452 |
| **cg22613010** | 0.027471 |
| **cg17338403** | 0.029662 |
| **cg04452713** | 0.034067 |
| **cg19273182** | 0.03497 |
| **cg24450312** | 0.042915 |
| **cg03286783** | 0.049725 |
| **cg06993413** | 0.180880 |
| **cg14424579** | 0.198533 |
| **cg14723032** | 0.218067 |
| **cg06926735** | 0.227325 |
| **cg03588357** | 0.305084 |
| **cg09646392** | 0.312717 |
| **cg07455279** | 0.424543 |
| **cg01968178** | 0.427676 |
| **cg16984944** | 0.436248 |
| **cg16241714** | 0.453502 |
| **cg25505610** | 0.488388 |
| **cg02047577** | 0.509941 |
| **cg01873645** | 0.608939 |
| **cg26297688** | 0.629988 |
| **cg00864867** | 0.718095 |
| **cg15703512** | 0.723257 |
| **cg25683012** | 0.813015 |
| **cg09441152** | 0.964471 |
| **cg00374717** | 0.988213 |
| **cg00436603** | 0.995672 |

**Table S6. Summary of stepwise regression for the first 28 CpG sites**

The markers contributing to the model are highlighted in green, while the ones in red are those that do not significantly change age prediction.

| **CpG sites** | **Step +in/-out** | **Multiple R** | **Multiple R^2^** | **R^2^ change** | **p value** |
| --- | --- | --- | --- | --- | --- |
| **cg22736354** | 1 | 0.835200 | 0.697559 | 0.697559 | 0.0000001 |
| **cg19761273** | 2 | 0.910351 | 0.828740 | 0.131181 | 0.0000001 |
| **cg20692569** | 3 | 0.920375 | 0.847090 | 0.018350 | 0.0000001 |
| **cg06493994** | 4 | 0.928244 | 0.861636 | 0.014547 | 0.0000001 |
| **cg27544190** | 5 | 0.939846 | 0.883311 | 0.021674 | 0.0000001 |
| **cg17274064** | 6 | 0.943643 | 0.890461 | 0.007151 | 0.0000001 |
| **cg04084157** | 7 | 0.946874 | 0.896571 | 0.006110 | 0.0000001 |
| **cg04528819** | 8 | 0.949625 | 0.901787 | 0.005216 | 0.0000001 |
| **cg01511567** | 9 | 0.951676 | 0.905687 | 0.003900 | 0.0000001 |
| **cg02085507** | 10 | 0.953522 | 0.909204 | 0.003517 | 0.0000001 |
| **cg07158339** | 11 | 0.954730 | 0.911510 | 0.002306 | 0.000001 |
| **cg05442902** | 12 | 0.956017 | 0.913968 | 0.002458 | 0.0000001 |
| **cg02479575** | 13 | 0.956845 | 0.915552 | 0.001583 | 0.000030 |
| **cg08370996** | 14 | 0.957458 | 0.916727 | 0.001175 | 0.000290 |
| **cg24450312** | 15 | 0.957983 | 0.917732 | 0.001005 | 0.000744 |
| **cg03286783** | 16 | 0.958845 | 0.919383 | 0.001652 | 0.000013 |
| **cg23092072** | 17 | 0.959382 | 0.920414 | 0.001030 | 0.000526 |
| **cg14308452** | 18 | 0.959720 | 0.921063 | 0.000650 | 0.005678 |
| **cg16408394** | 19 | 0.959903 | 0.921414 | 0.000350 | 0.041706 |
| **cg24126851** | 20 | 0.960074 | 0.921742 | 0.000329 | 0.048114 |
| **cg04452713** | 21 | 0.960309 | 0.922194 | 0.000452 | 0.020294 |
| **cg22613010** | 22 | 0.960498 | 0.922556 | 0.000362 | 0.037302 |
| **cg17338403** | 23 | 0.960724 | 0.922990 | 0.000434 | 0.022426 |
| **cg19273182** | 24 | 0.960791 | 0.923119 | 0.000129 | 0.212676 |
| **cg04999691** | 25 | 0.960858 | 0.923247 | 0.000129 | 0.213073 |
| **cg06993413** | 26 | 0.960937 | 0.923400 | 0.000153 | 0.170420 |
| **cg06926735** | 27 | 0.961028 | 0.923575 | 0.000174 | 0.146738 |
| **cg14723032** | 28 | 0.961108 | 0.923728 | 0.000154 | 0.172929 |

**Table S7. Pearson correlations between all CpG markers used in the optimised GRNN model and age**

| **Variables** | **Age** | **cg19761273** | **cg27544190** | **cg03286783** | **cg01511567** | **cg07158339** | **cg05442902** | **cg24450312** | **cg17274064** | **cg02085507** | **cg20692569** | **cg04528819** | **cg08370996** | **cg04084157** | **cg22736354** | **cg06493994** | **cg02479575** |
| --- | --- | --- | --- | --- | --- | --- | --- | --- | --- | --- | --- | --- | --- | --- | --- | --- | --- |
| **Age** | 1 |  |  |  |  |  |  |  |  |  |  |  |  |  |  |  |  |
| **cg19761273** | -.682^**^ | 1 |  |  |  |  |  |  |  |  |  |  |  |  |  |  |  |
| **cg27544190** | -.338^**^ | .601^**^ | 1 |  |  |  |  |  |  |  |  |  |  |  |  |  |  |
| **cg03286783** | .072^*^ | .197^**^ | .509^**^ | 1 |  |  |  |  |  |  |  |  |  |  |  |  |  |
| **cg01511567** | -.422^**^ | .585^**^ | .555^**^ | .335^**^ | 1 |  |  |  |  |  |  |  |  |  |  |  |  |
| **cg07158339** | -.504^**^ | .462^**^ | .266^**^ | .065^*^ | .370^**^ | 1 |  |  |  |  |  |  |  |  |  |  |  |
| **cg05442902** | -.408^**^ | .616^**^ | .577^**^ | .323^**^ | .578^**^ | .369^**^ | 1 |  |  |  |  |  |  |  |  |  |  |
| **cg24450312** | .082^**^ | .000 | .144^**^ | .271^**^ | -.018 | -.144^**^ | .144^**^ | 1 |  |  |  |  |  |  |  |  |  |
| **cg17274064** | -.283^**^ | .429^**^ | .436^**^ | .381^**^ | .498^**^ | .394^**^ | .397^**^ | -.184^**^ | 1 |  |  |  |  |  |  |  |  |
| **cg02085507** | .345^**^ | -.109^**^ | -.050 | -.036 | -.113^**^ | -.104^**^ | -.088^**^ | .097^**^ | -.071^*^ | 1 |  |  |  |  |  |  |  |
| **cg20692569** | .706^**^ | -.407^**^ | -.164^**^ | .138^**^ | -.216^**^ | -.340^**^ | -.153^**^ | .173^**^ | -.177^**^ | .298^**^ | 1 |  |  |  |  |  |  |
| **cg04528819** | .698^**^ | -.371^**^ | -.016 | .282^**^ | -.149^**^ | -.257^**^ | -.160^**^ | -.017 | .039 | .306^**^ | .524^**^ | 1 |  |  |  |  |  |
| **cg08370996** | .614^**^ | -.127^**^ | .268^**^ | .457^**^ | .072^*^ | -.215^**^ | .089^**^ | .131^**^ | .188^**^ | .264^**^ | .534^**^ | .650^**^ | 1 |  |  |  |  |
| **cg04084157** | .664^**^ | -.227^**^ | .103^**^ | .397^**^ | .016 | -.211^**^ | .038 | -.029 | .170^**^ | .188^**^ | .545^**^ | .611^**^ | .728^**^ | 1 |  |  |  |
| **cg22736354** | .796^**^ | -.347^**^ | .020 | .340^**^ | -.068^*^ | -.299^**^ | -.056 | .042 | .042 | .239^**^ | .648^**^ | .690^**^ | .771^**^ | .802^**^ | 1 |  |  |
| **cg06493994** | .626^**^ | -.179^**^ | .166^**^ | .417^**^ | .104^**^ | -.150^**^ | .048 | -.225^**^ | .247^**^ | .162^**^ | .481^**^ | .689^**^ | .761^**^ | .805^**^ | .838^**^ | 1 |  |
| **cg02479575** | .294^**^ | -.021 | .410^**^ | .690^**^ | .119^**^ | -.140^**^ | .147^**^ | .464^**^ | .177^**^ | .136^**^ | .269^**^ | .371^**^ | .568^**^ | .397^**^ | .407^**^ | .360^**^ | 1 |

**Table S8. Co-linearity Statistics for all variables in the GRNN model**

| **Variables** | **Unstandardized Coefficients** | | **Standardized Coefficients** | **t** | **Sig.** | **Collinearity Statistics** | |
| --- | --- | --- | --- | --- | --- | --- | --- |
|  | **B** | **Std. Error** | **Beta** |  |  | **Tolerance** | **VIF** |
| **Age** | 44.353 | 2.915 |  | 15.216 | .000 |  |  |
| **cg19761273** | -92.570 | 6.939 | -.198 | -13.341 | .000 | .348 | 2.876 |
| **cg27544190** | -97.817 | 12.114 | -.114 | -8.075 | .000 | .381 | 2.628 |
| **cg03286783** | -68.945 | 12.903 | -.077 | -5.343 | .000 | .371 | 2.696 |
| **cg01511567** | -38.106 | 7.034 | -.067 | -5.418 | .000 | .493 | 2.030 |
| **cg07158339** | -22.690 | 4.648 | -.053 | -4.881 | .000 | .658 | 1.520 |
| **cg05442902** | -53.958 | 8.110 | -.085 | -6.653 | .000 | .470 | 2.128 |
| **cg24450312** | 31.821 | 5.951 | .071 | 5.347 | .000 | .436 | 2.293 |
| **cg17274064** | -54.867 | 7.065 | -.092 | -7.766 | .000 | .546 | 1.833 |
| **cg02085507** | 23.746 | 2.830 | .081 | 8.390 | .000 | .820 | 1.219 |
| **cg20692569** | 32.367 | 4.629 | .089 | 6.992 | .000 | .469 | 2.133 |
| **cg04528819** | 50.618 | 7.149 | .099 | 7.080 | .000 | .390 | 2.564 |
| **cg08370996** | 58.048 | 11.444 | .089 | 5.072 | .000 | .245 | 4.077 |
| **cg04084157** | 76.679 | 9.726 | .131 | 7.884 | .000 | .276 | 3.618 |
| **cg22736354** | 82.406 | 6.324 | .283 | 13.031 | .000 | .162 | 6.172 |
| **cg06493994** | 49.744 | 9.255 | .131 | 5.375 | .000 | .129 | 7.743 |
| **cg02479575** | 53.739 | 16.594 | .049 | 3.238 | .001 | .327 | 3.059 |

**Supplementary Figures:**

**Figure S1. Age distribution of samples used in the age prediction model (n=1,156)**


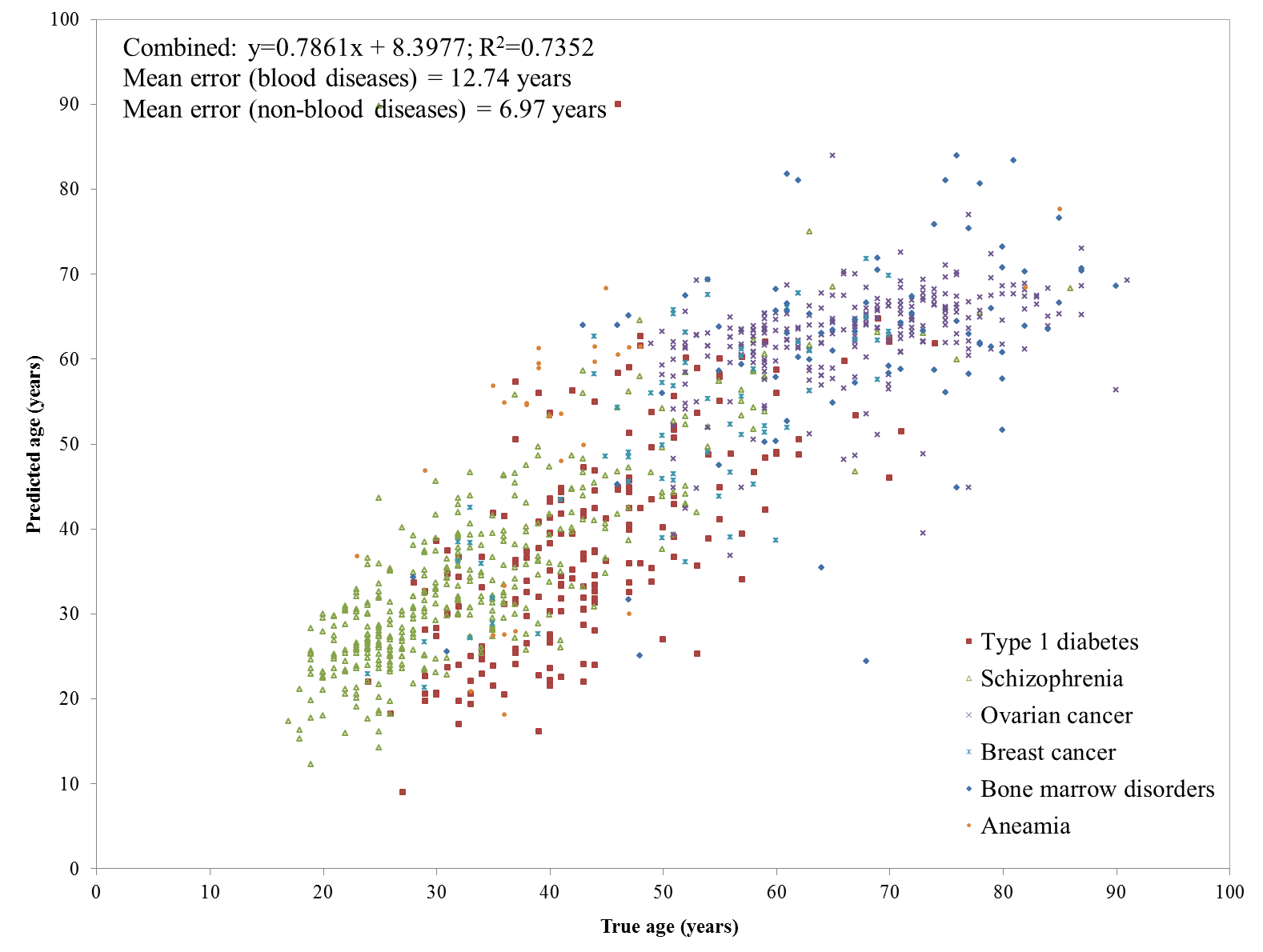


**Figure S2. Age predictions in whole blood samples from volunteers with various diseased states (n=1,011)**


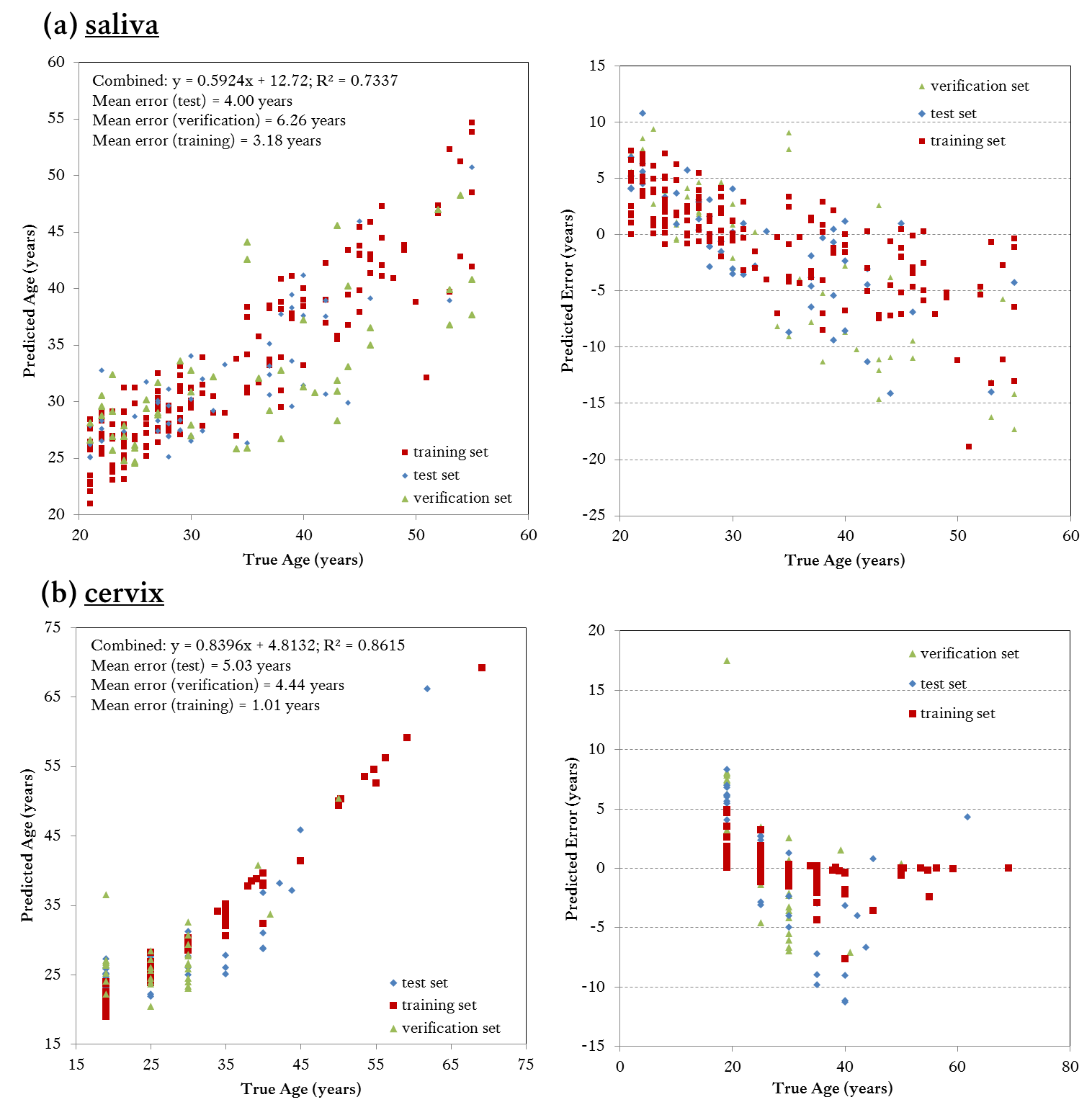


**Figure S3. Age predictions in saliva samples (n=265)**
